# Varying Presentations of Multisystem Inflammatory Syndrome Temporarily Associated with COVID-19

**DOI:** 10.1155/2020/8878946

**Published:** 2020-11-23

**Authors:** Jasmina Krikilion, Lisa Nuyttens, Siel Daelemans, Karlien François, Reiner Mauel, Daniel De Wolf, Gerlant van Berlaer

**Affiliations:** ^1^Vrije Universiteit Brussel (VUB), Universitair Ziekenhuis Brussel (UZ Brussel), Department of Paediatrics, Brussel, Belgium; ^2^Vrije Universiteit Brussel (VUB), Universitair Ziekenhuis Brussel (UZ Brussel), Division of Nephrology and Hypertension, Brussel, Belgium; ^3^Vrije Universiteit Brussel (VUB), Universitair Ziekenhuis Brussel (UZ Brussel), Paediatric Emergency and Intensive Care Unit, Brussel, Belgium

## Abstract

*Background*. A novel coronavirus identified in 2019 leads to a pandemic of severe acute respiratory distress syndrome with important morbidity and mortality. Initially, children seemed minimally affected, but there were reports of cases similar to (atypical) Kawasaki disease or toxic shock syndrome, and evidence emerges about a complication named paediatric inflammatory multisystem syndrome temporarily associated with SARS-CoV-2 (PIMS-TS) or multisystem inflammatory syndrome in children (MIS-C). *Case Presentations.* Two cases were compared and discussed demonstrating varying presentations, management, and evolution of MIS-C. These cases are presented to increase awareness and familiarity among paediatricians and emergency physicians with the different clinical manifestations of this syndrome. *Discussion.* MIS-C may occur with possible diverse clinical presentations. Early recognition and treatment are paramount for a beneficial outcome.

## 1. Introduction

In December 2019, a novel coronavirus was identified in Wuhan (China) as the cause of an atypical pneumonia which may cause severe acute respiratory distress syndrome. The virus rapidly spread around the world and became a global pandemic. The virus was named “severe acute respiratory syndrome coronavirus 2 (SARS-CoV-2)” by the World Health Organization. The disease has been termed “COVID-19” [1].

In Belgium, the first case was diagnosed in February 2020. During the following months, more information became available about the course of the disease. In children, a SARS-CoV-2 infection is usually asymptomatic or mild [2]. However, more reports emerged of a presentation in children similar to Kawasaki disease or toxic shock syndrome [3, 4]. The syndrome has been named multisystem inflammatory syndrome in children (MIS-C), paediatric multisystem inflammatory syndrome, paediatric inflammatory multisystem syndrome temporarily associated with SARS-CoV-2 (PIMS-TS), or paediatric hyperinflammatory syndrome [5] and has been defined by the CDC (see Table 1) and the WHO (see Table 2) [6, 7].

In our tertiary teaching hospital, we detected two cases of MIS-C that are worthwhile comparing, discussing, and sharing with the wider paediatric community, in order to optimize early recognition and management of MIS-C.

Both patients provided informed consent, and approval of the Ethical Committee was granted.

## 2. Case Presentation 1

The first case concerns a 15-year-old boy, known with idiopathic end-stage kidney disease (ESKD) treated with peritoneal dialysis. He had no history of cardiac dysfunction. He was admitted on March 6^th^, 2020, for a deceased-donor kidney transplantation which was postponed because of fever for 3 days. He complained of fatigue, headaches, and loss of appetite. Characteristics and laboratory findings at admission and imaging results are noted in Table 3. A positive PCR for SARS-CoV-2 confirmed COVID-19 the day of admission. He was hospitalized for 10 days because isolation measures were not possible at home. He stayed paucisymptomatic and received supportive care with antipyretics besides his usual treatments for kidney failure. After 10 days, he was discharged home.

Twenty-three days after discharge, he was readmitted because of dyspnea (NYHA grade III), orthopnea, and hemoptysis. Laboratory findings and imaging results are shown in Table 3. Because of suspected alveolar hemorrhage, bronchoscopy was performed the day after admission. Repeated bronchoalveolar lavage ruled out alveolar bleeding. SARS-CoV-2 PCR on lower respiratory samples was negative. Because of progressive oxygen requirements after bronchoscopy and severe myocardial dysfunction on echocardiography, the patient was transferred to the intensive care unit for monitoring, inodilator therapy, and noninvasive ventilation (NIV) with rapid clinical recovery. NIV could be stopped, and supplemental oxygen was given by high-flow nasal cannula until day 4 of hospitalization. Endomyocardial biopsy was performed, but neither endomyocardial PCR nor histopathological analysis was suggestive for SARS-CoV-2 myocarditis. Cardiac MRI on day 8 revealed a dilated left ventricle with reduced ejection fraction of 30–35%. Coronarography revealed normal coronal anatomy.

While titrating heart failure therapy, he needed readmission in the medium care unit from day 9 to 12 because of an episode of relapse cardiac decompensation that needed to be managed by increasing ultrafiltration through peritoneal dialysis.

Between day 18 and 20, he developed a low-degree fever (maximum body temperature of 38.7°C axillary). C-reactive protein was low, and lymphocytosis was absent. Because of the suspicion of pneumonia, co-amoxiclav was empirically started. All cultures (blood, sputum, peritoneal fluid, and urine) yielded negative. Because of nocturnal hypoxemia, a polysomnography was performed revealing Cheyne–Stokes apneas secondary to heart failure. After 28 days, he was ready for a new discharge home. He is still under medical follow-up for his end-stage kidney disease and slowly recovering heart failure.

This patient was one of the first cases of paediatric SARS-CoV-2 infection in Belgium. In retrospect, we think he was one of the first cases of a MIS-C associated with a SARS-CoV-2 infection in Europe, with signs of viral or inflammatory myocarditis. At that time, little was known about the treatment of a MIS-C associated with COVID-19 infection, so no immunoglobulins or corticoids were administered during his stay.

## 3. Case Presentation 2

The second case, a girl of 14 years old, known with Lennox–Gastaut syndrome, was admitted on April 28^th^, 2020, in our hospital for neurological observation after a severe concussion. The second day of admission, she developed high fever initially not responding to antipyretics, and she became generally unwell with a dry cough. The patient and her mother had COVID-19-like symptoms approximately a month prior to admission; unfortunately, no confirmation with PCR-testing for SARS-CoV-2 was obtained at that time. Additional characteristics, laboratory findings, and chest X-ray results are shown in Table 3.

Epileptic activity increased, and she needed seizure medication. Because of seizures and the risk of aspiration pneumonia, intravenous co-amoxiclav was empirically initiated. She gradually deteriorated needing oxygen therapy. Because fever persisted and inflammation parameters were rising, intravenous antibiotic therapy was switched to third generation cephalosporins. Cardiac ultrasound, ECG, and echocardiography were planned, as well as a chest CT which all showed signs of pericarditis (see Table 3).

**Figure 1:**
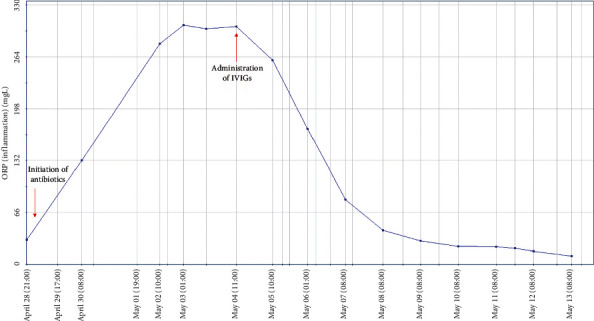
Course of C-reactive protein in Case 2 in the year 2020.

Our patient did not meet the criteria for Kawasaki disease but appeared to be an atypical Kawasaki-like shock syndrome. A trial of intravenous immunoglobulins (IVIGs) was administered. Although none of the cultures (blood, cerebrospinal fluid, urine, stool, and bronchial aspirates) yielded positive, antibiotics were continued for 10 days. Shortly after IVIG treatment, the patient clinically improved significantly although moderate fever persisted and inflammatory parameters dropped swiftly (as visualized in Figure 1). Oxygen therapy was interrupted, and all edema disappeared. In the light of clinical and laboratory improvement to normalization, there seemed no need for additional corticotherapy.

After hospital discharge, SARS-CoV-2 serology returned positive and diagnosis of MIS-C associated with COVID-19 was confirmed. She remains under medical follow-up for her epilepsy but recovered completely from PIMS-TS.

## 4. Discussion

Multisystem inflammatory syndrome in children (MIS-C) is an uncommon entity. During this global pandemic with SARS-CoV-2, some paediatric cases emerged demonstrating a formerly unknown complication of COVID-19, meeting criteria of typical or atypical Kawasaki disease, toxic shock syndrome, and macrophage activation syndrome [3, 8, 9]. Our cases fulfilled the definition of MIS-C, as described by the Centre for Disease Control (CDC) in the USA [6] or by the World Health Organization (WHO) [7].

It is noteworthy that these new developing cases are generally older (teenagers) than the typical Kawasaki patient, who is generally younger than six years old.

Comparing the two cases in this study with other cases described in the literature, we note that MIS-C is mostly described in patients with following characteristics: young adolescents, African origin, and BMI >25 kg/m^2^ [3, 8, 10]. Our cases absolutely match those characteristics. Both cases were young teens of African origin; patient 1 had a normal BMI, whereas patient 2 had a BMI above 95^th^ percentile. Although the basic features are similar in our two patients, there is a variation in clinical presentation: Case 1 presented with respiratory distress and heart failure, whereas patient 2 presented with symptoms similar to toxic shock syndrome. In the literature, a wide variety of clinical presentations is described although they have the same underlying entity [3, 8]. Pathogenesis is not yet fully understood.

Early recognition of MIS-C is paramount for a beneficial outcome, but recognition is complicated by the wide variety in initial clinical presentation and difficulties in interpretation of additional investigations. During the COVID-19 pandemic, high suspicion should be raised in every child presenting with signs of sepsis or vasculitis. In those children, additional investigations to confirm infection with SARS-CoV-2 are indicated; however, they are not always sensitive: with a sensitivity of only 71–98%, a negative PCR for SARS-CoV-2 does not rule out a SARS-CoV-2 infection [3, 9, 11]. Patient 2 had a negative PCR twice, but serology confirmed diagnosis. This patient did have a possible COVID-19 exposure a few weeks prior to admission. As described in the literature, MIS-C can occur in the acute phase of COVID-19 but also as a postinflammatory syndrome [12]. Although chest CT in acute COVID-19 in adults is very sensitive [13], imaging of the chest in children with MIS-C can be normal [8, 10]. Close cardiac monitoring is warranted although cardiac ultrasound does not always reveal abnormalities [8, 10]. Blood sampling of cardiac enzymes should be done in every child with suspicion of MIS-C [4]. This often shows elevation of D-dimers, NT-proBNP, troponins, and fibrinogen, as seen in our first case (fibrinogen in the second case was not tested in the acute phase of disease) [14]. These laboratory results are not often seen in typical Kawasaki which can help guide diagnosis.

In treating MIS-C, a multidisciplinary approach is desirable as all organ systems can be involved and associated morbidity and mortality is described in the literature [3, 8].

Antibiotic therapy is often initiated because of the clinical resemblance to toxic shock syndrome and sepsis and the high inflammatory parameters. Both patients in this study were empirically started on antibiotics as per sepsis protocol, but in both cases all cultures yielded negative. Thus, in suspicion of MIS-C, antibiotics can be stopped early if cultures stay negative after 48 hours.

In MIS-C, imminent treatment with intravenous immunoglobulins is associated with a beneficial outcome. In our cases, patient 2 showed swift improvement with IVIG alone. Case 1 did not receive any treatment other than supportive treatment. We can only speculate that additional treatment might have improved his course of disease and length of hospital stay. Suggested management in other case reports includes intravenous immunoglobulins or corticoids [3, 12]. In one case report, the patient was treated with tocilizumab, an IL-6 receptor antagonist [10]. A study published in April 2020 showed significant clinical improvement after administration of tocilizumab to patients with COVID-19 pneumonia associated with acute respiratory distress syndrome [9].

We are convinced that sharing the features in this subset of children will enhance the awareness for MIS-C in the wider paediatric community and may help to optimize early recognition and management of this novel syndrome.

## Figures and Tables

**Table 1:** Definition of MIS-C by the CDC [6].

(1) An individual aged <21 years presenting with fever, laboratory evidence of inflammation, and evidence of clinically severe illness requiring hospitalization, with multisystem (>2) organ involvement (cardiac, renal, respiratory, hematologic, gastrointestinal, dermatologic, or neurological)
(2) AND no alternative plausible diagnoses
(3) AND positive for current or recent SARS-CoV-2 infection by RT-PCR, serology, or antigen test, or COVID-19 exposure within the 4 weeks prior to the onset of symptoms

**Table 2:** Definition of MIS-C by the WHO [7].

(1) Children and adolescents 0–19 years of age with fever >3 days
(2) AND two of the following:
(i) Rash or bilateral nonpurulent conjunctivitis or mucocutaneous inflammation signs (oral, hands, or feet)
(ii) Hypotension or shock
(iii) Features of myocardial dysfunction, pericarditis, valvulitis, or coronary abnormalities (including ECHO findings or elevated troponin/NT-proBNP)
(iv) Evidence of coagulopathy (by PT, PTT, and elevated D-dimers)
(v) Acute gastrointestinal problems (diarrhoea, vomiting, or abdominal pain)
(3) AND elevated markers of inflammation such as ESR, C-reactive protein, or procalcitonin
(4) AND no other obvious microbial cause of inflammation, including bacterial sepsis and staphylococcal or streptococcal shock syndromes
(5) AND evidence of COVID-19 **(**RT-PCR, antigen test, or serology positive) or likely contact with patients with COVID-19

**Table 3:** Comparing clinical signs and symptoms, lab and imaging, and treatment and outcome for two cases of MIS-C.

	Case 1		Case 2
*Characteristics*			
Gender	Male		Female
Age (years)	15		14
	*1* ^*st*^ *admission*	*2* ^*nd*^ *admission*	
Weight (kg)	60	67	50
BMI (kg/m^2^)	19.59	21.88	34.72

*Signs and symptoms*			
Fever	Yes	No	Yes
Cough, dyspnea	Yes	Yes	Yes
Sore throat	No	No	Yes
Decreased appetite	No	Yes	Yes
Nausea, vomiting	No	No	No
Diarrhoea	No	No	Yes
Fatigue	Yes	Yes	Yes
Headache	Yes	No	Yes
Rash	Yes	No	Yes
Desaturation <92%	No	Yes	Yes
Edema	No	Yes	Yes

*PICU referral*			
Reason of referral	No PICU referral	Respiratory deterioration	Respiratory distress and excessive edema with early signs of shock
Organ support	None	NIV	Oxygen therapy through nasal cannula
Pharmacological treatment	None	Vasopressins, antibiotics, diuretics, morphin	Antibiotics, antipyretics, diuretics, intravenous immunoglobulins
LOS in hospital	10	28	23
LOS in PICU	0	7	10

*Imaging result*			
Chest X-ray	Normal	Bilateral consolidations	Consolidation left upper lobe
Chest CT	No	Bilateral consolidations	Pericarditis and bilateral consolidations
Heart ultrasound	No	(i) Dilated left ventricle, ejection fraction 30–35%. (ii) Diastolic dysfunction grade 1 (iii) Normal right ventricle (iv) Dilated left atrium (v) MI 2/4, TI 1/4, dilated truncus pulmonalis (vi) Pericardial effusion (5 mm), no hemodynamical consequences	(i) Pericarditis with pericardial effusion 15 mm, no hemodynamical consequences
ECG	Normal	Negative T-waves in the anterior wall with prolonged QT-segment (534 ms)	Peripheral low voltages with flattened T-waves in left precordials

*Lab results*	(i) Lymphopenia (929/mm³) (ii) Thrombocytopenia (116.000/mm³) (iii) CRP 30 mg/L (maximum) (iv) Albumin 31 g/L (v) Normal hepatic enzymes	(i) No lymphopenia/leukopenia (ii) No thrombocytopenia or thrombocytosis (iii) Anemia: hemoglobin 7.3 g/dL (iv) CRP 56.6 mg/L (v) Albumin 26 g/L (vi) Normal hepatic enzymes (vii) Cardiac enzymes: (a) NT-proBNP 155919.0 ng/L (b) Cardiac troponin *T* 0.314 µg/L (viii) D-dimer 6943 ng/ml (ix) Fibrinogen 482 mg/dL	(i) Lymphopenia (267/mm³) (ii) Thrombocytosis (808.000/mm³) (iii) Hemoglobin 7.6 g/dL (iv) CRP 304.3 mg/L (maximum) (v) Albumin 24 g/L (vi) Cardiac enzymes: (a) NT-proBNP 3228.0 ng/L (b) Cardiac troponin 0.01 mcg/L (vii) D-dimer 3584 ng/ml (viii) Fibrinogen 322 mg/dL

*Microbiology results*	(i) Nasopharyngeal swab: PCR SARS-CoV-2 positive	(i) Nasopharyngeal swab: PCR SARS-CoV-2 negative (ii) Heart biopsy: PCR SARS-CoV-2 negative (iii) SARS-CoV-2 IgG ELISA positive (54.30 AE/mL)	(i) Nasopharyngeal swab: PCR SARS-CoV-2 negative (twice) (ii) SARS-CoV-2 IgG ELISA positive (41.30 AE/mL)

## Data Availability

All data generated or analyzed during this study are included in this published article. Additional data are available upon simple request to the corresponding author. All authors confirm that they had full access to all of the data in the study and they can take responsibility for the integrity of the data and the accuracy of the data analysis.

## Abbreviations

BMI: Body mass index
CDC: Centre for Disease Control (USA)
COVID-19: Coronavirus disease-19
CRP: C-reactive protein
CT: Computer tomography
ECG: Electrocardiogram
ESKD: End-stage kidney disease
IVIG: Intravenous immunoglobulins
MIS-C: Multisystem inflammatory syndrome in children
MRI: Magnetic resonance imaging
NIV: Noninvasive ventilation
NT-proBNP: N-terminal pro-brain natriuretic peptide
PCR: Polymerase chain reaction
PIMS-TS: Paediatric inflammatory multisystem syndrome temporarily associated with SARS-CoV-2
SARS-CoV-2: Severe acute respiratory syndrome coronavirus type 2.
